# Data management (and sharing) in neuroscience: balancing possible, practical and perfect solutions

**DOI:** 10.1093/braincomms/fcae450

**Published:** 2024-12-24

**Authors:** Tara L Spires-Jones

**Affiliations:** Edinburgh

## Abstract

Our editor discusses re-organizing her lab’s data storage to facilitate sharing and archiving data. She also advertises the ‘Brain Communications’ early career researcher paper prize for the first author of a paper published in the journal in 2024—please send nominations!

## Graphical Abstract

**Graphical abstract:**
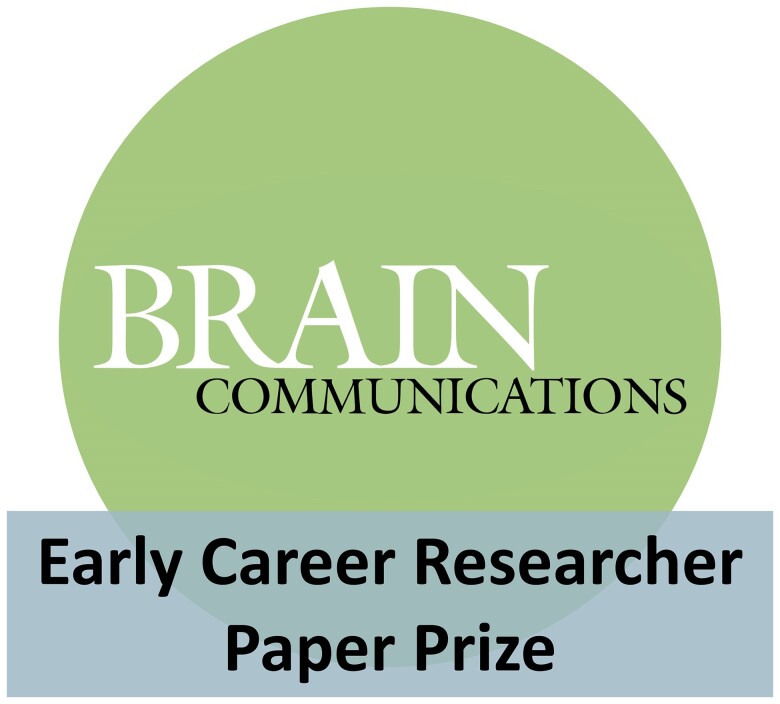

Welcome to Volume 7, issue 1 of *Brain Communications*. Recently in my lab group, we have re-organized our data storage on our server to institute a standard file naming and organization system that all lab members are required to use. After struggling to come up with a good solution and a finding very little practical advice on how to do this, I decided to share our system in case it can save anyone else time. I hope this will be particularly useful for people starting their labs as it would have been much easier to start from scratch than to re-organize years’ worth of data into a new system!

The unenviable task of data re-organization was motivated by several recurring issues. One was that after people leave the lab, it can be next to impossible to interpret their file-naming system to find raw data. Please do not judge my lack of organizational skills, but we previously had a system of each person having a folder on the server to organize as they wished, although there was a firm rule that all data had to be kept on the shared lab server. We then collected all raw data for each figure in a published paper into a folder after publication to organize everything for easy data sharing. However, as most of you will know, not every single piece of data is published, and this task of collating data from folders of different lab members who collected the data was quite painful, particularly if they had already left the lab and had convoluted folder naming conventions—think ‘side project 2’ or ‘non-dissertation experiments’ as examples of non-informative folder names. So I decided to organize our files in a way that makes it easy to locate raw data, analysed data, etc. I had long think about the best way to do this and searched around for examples of best practice. There is remarkably little out there. While we are all required to write data management plans, there is very little in terms of the nitty-gritty of the most efficient ways to organize data. Kanza and Knight advised using ‘sensible file structures that have been agreed with the entire team’.^1^ Some colleagues recommended that data be organized by ‘project’ and in previous iterations of organization I had tried this. But projects are fluid. I thought about organizing by grant, but many projects continue with different funding or change focus. Organizing by lab members did not work for me (see above regarding finding files).

In the end, we decided on a structure that is organized by type of data and then by date (see Fig. 1). Within each type of data, we have a standard file-naming structure. In our microscopy raw data folder, people add new data as it is collected by creating a new folder starting with the date, followed by the initials of the person taking the data, and then a description of the experiment. Within each experiment, there is a standard file-naming convention for each data type. In microscopy this is the subject ID—brain region—sample ID—channel description. We have files in the root directory of the server explaining how to name files and folders and, in bold red text, the reminder that all data MUST MUST MUST be stored by these conventions on our backed-up, university-managed server at the time of collection and analysis.

**Figure 1:**
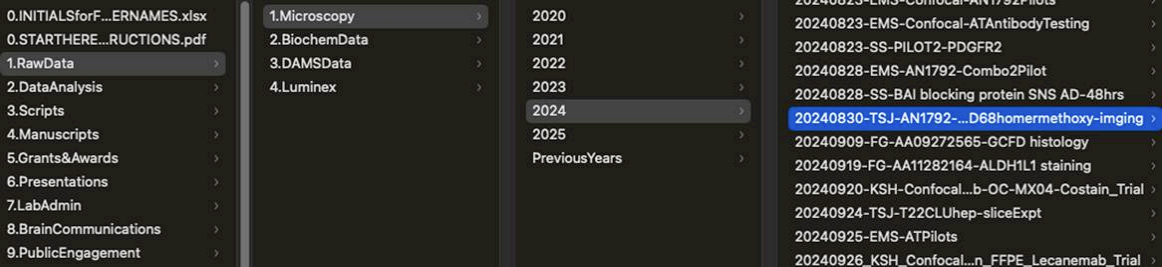
Screenshot of our file structure.

The other pillar of our data management strategy in the lab is our electronic lab notebook. Every experiment is recorded in the lab notebook, including the file path to the server location where raw data are stored. I cannot over-emphasize how amazing the electronic lab notebook is as the leader of a lab team. I can help people design experiments by directly checking their detailed protocols in the lab notebook. Multiple people can collaborate easily on data collection by adding to the same lab notebook entry as the experiment progresses. And when it is time to write the paper, it is very easy to write the methods following along from the online protocol and to find where raw data are through the server path in the lab notebook entry.

In addition to generally better organization and saving my sanity when looking for files, another motivation for the grand re-organization was running out of server space on the 1 PB departmental server supported by our institute’s Information Services department. Before buying the server, we asked all principle investigators to estimate their data storage needs over a 5-year period and we all wildly underestimated. This prompted me to think about whether we really needed every single bit of data ever collected over my 18 years as principal investigator on an active server (OK, probably not, but I hoard data). We have an archive tape server at our university but it is amazingly impractical and expensive to use. While it is physically painful to delete data, I have been thinking recently about not only the cost but also the sustainability issues around data storage, which does use energy. It is estimated that around 2% of global greenhouse gas emissions are due to computing, and as a sector, neuroscientists are starting to think about the best ways to manage our data to reduce our carbon footprint. See, for example, the 10 tips from Souter *et al*.^2^ for reducing carbon footprint in neuroimaging studies.

A key motivation for wanting to keep data is to be able to share raw data if anyone wants to re-use the data for their own research. However, our raw data associated with publications are rarely used. While I am a strong believer in open science and share every spreadsheet and statistics file associated with our papers on a publicly available server (https://datashare.ed.ac.uk/handle/10283/3076), it is not practical for my group to upload every single raw data file with associated metadata to make them findable, accessible, interpretable and reusable in line with the FAIR principle.^3^ Our group largely takes microscope images and analyses them. Annotating and uploading all of these for every study would take a huge amount of time and money to organize, and although we have been asked for spreadsheets of data, I have never once been asked for raw images. In an ideal world, I would be able to afford to pay someone to spend the months of time it would take to fully annotate our data and pay to share it on servers; however, I do not have that funding and it seems a waste of energy since literally no one has asked me for raw images in 18 years other than in collaborative projects.

Keeping raw data is also motivated by being able to defend our work if we are ever accused of misconduct. We try very hard as a group to conduct rigorous work and be transparent about our processes but mistakes can happen and I want to be sure we have the raw data and lab notebook entries to be able to go back to if there are mistakes or any accusations. But again, this will hopefully be a rare event. In the past, a collaborating lab was accused of fraud due to copy-paste errors in figure preparation during paper revisions, and we had to go back through paper lab notebooks and individual computer files to clear it up, but thankfully nothing has happened since then.

Balancing these factors, I have decided to keep all raw data for 5 years and hopefully archive data older than that, deleting many files that are intermediate versions used for analyses. We are still working on the best way to archive data so any tips are welcome! If anyone would like more details about how we organize our data, I would be very happy to share.

At *‘Brain Communications’*, we encourage data sharing and require sharing of code associated with our papers. We also encourage rigorous experimental design through requiring thorough statistical reporting and checking for a few common statistical faux pas in our papers (e.g. lack of correction for multiple comparisons). We also publish registered reports, which allow people to have their experimental design peer-reviewed before completing the study. See, for example, this report from Willis *et al*.^4^ detailing their plan to test visual training in people with visual field deficits after stroke, which was reviewed and accepted in 2020. When the study was completed in 2024, the group published the Stage 2 registered report showing that visual discrimination training in the blind part of the visual field improved vision.^5^

On another topic, as it is the new year, I hope you will consider nominating the first author of a ‘Brain Communications’ paper published in 2024 for our Early Career Researcher Paper Prize. To nominate an Early Career Researcher first author (student, postdoc or technician) please fill in this short nomination form by the end of January https://forms.gle/pydjm2FLVBJYUaNq5. The winner will be invited to receive the prize and give a short talk at the Brain Conference in person on March 7, 2025 in https://conference.guarantorsofbrain.org/, so please confirm with the nominee that they would be able to attend before submitting the nomination. Our editorial board members will vote and the winner will be announced in February 2025.

The cover image for this issue comes from Bureau *et al*.^6^ and is a transmission electron microscopy image showing mitochondrial fragmentation, increased mitophagy, and protein aggregation in the prelaminar optic nerve of a presymptomatic Opa1+/− mouse.
